# A novel quantitative prognostic model for initially diagnosed non-small cell lung cancer with brain metastases

**DOI:** 10.1186/s12935-022-02671-2

**Published:** 2022-08-11

**Authors:** Xiaohui Li, Wenshen Gu, Yijun Liu, Xiaoyan Wen, Liru Tian, Shumei Yan, Shulin Chen

**Affiliations:** 1 State Key Laboratory of Oncology in South China, Collaborative Innovation Center for Cancer Medicine, Guangzhou, 510060 People’s Republic of China; 2 Department of Clinical Laboratory Medicine, Sun Yat-Sen University Cancer Center, Guangzhou, 510060 People’s Republic of China; 3 Department of Laboratory Medicine, Guangdong Provincial Key Laboratory of Malignant Tumor Epigenetics and Gene Regulation, Sun Yat-Sen Memorial Hospital, Guangzhou, 510120 People’s Republic of China; 4 Department of Central Sterile Supply, Guanghua School of Stomatology, Affiliated Stomatological Hospital, Guangdong Province Key Laboratory of Stomatology, Sun Yat-Sen University, Guangzhou, Guangdong 510055 People’s Republic of China; 5 Research Center for Translational Medicine, the First Affiliated Hospital, Sun Yat-Sen University, 58 Zhongshan Road 2, Guangzhou, Guangdong 510080 People’s Republic of China; 6 Department of Pathology, Sun Yat-Sen University Cancer Center, Guangzhou, 510060 People’s Republic of China; 7 Guangdong Esophageal Cancer Institute, Guangzhou, 510060 People’s Republic of China

**Keywords:** Brain metastases, Non-small cell lung cancer, Prognostic model, Serum inflammatory indexes, LASSO-Cox regression analysis

## Abstract

**Background:**

The prognosis of non-small cell lung cancer (NSCLC) with brain metastases (BMs) had been researched in some researches, but the combination of clinical characteristics and serum inflammatory indexes as a noninvasive and more accurate model has not been described.

**Methods:**

We retrospectively screened patients with BMs at the initial diagnosis of NSCLC at Sun Yat-Sen University Cancer Center. LASSO-Cox regression analysis was used to establish a novel prognostic model for predicting OS based on blood biomarkers. The predictive accuracy and discriminative ability of the prognostic model was compared to Adjusted prognostic Analysis (APA), Recursive Partition Analysis (RPA), and Graded Prognostic Assessment (GPA) using concordance index (C-index), time-dependent receiver operating characteristic (td-ROC) curve, Decision Curve Analysis(DCA), net reclassification improvement index (NRI), and integrated discrimination improvement index (IDI).

**Results:**

10-parameter signature's predictive model for the NSCLC patients with BMs was established according to the results of LASSO-Cox regression analysis. The C-index of the prognostic model to predict OS was 0.672 (95% CI = 0.609 ~ 0.736) which was significantly higher than APA,RPA and GPA. The td-ROC curve and DCA of the predictive model also demonstrated good predictive accuracy of OS compared to APA, RPA and GPA. Moreover, NRI and IDI analysis indicated that the prognostic model had improved prediction ability compared with APA, RPA and GPA.

**Conclusion:**

The novel prognostic model demonstrated favorable performance than APA, RPA, and GPA for predicting OS in NSCLC patients with BMs.

**Supplementary Information:**

The online version contains supplementary material available at 10.1186/s12935-022-02671-2.

## Introduction

According to research, almost one third of non-small cell lung cancer (NSCLC) patients will have brain metastases (BMs) and it constitutes the most common source of BMs [1], and the median survival time is only 1 month if without effective treatment [2]. BMs are the most common malignant brain tumors in adults. Over the past decade, the incidence of BMs has increased due to improvements in the diagnosis and systematic treatment of extracranial diseases [3]. However, it is difficult to treat BMs with systemic chemotherapy since the agents have difficulty crossing the blood–brain barrier. Therefore, it is important to estimate the patient's survival prognosis.

Some models that have been reported to predict the prognosis of patients with BMs include Recursive Partition Analysis (RPA) [4, 5], the Score Index for Radiosurgery (SIR) [6], Basic Score for Brain Metastases(BSBM) [7] and Graded Prognostic Assessment (GPA) [8]. Nowadays, the most widely used prognostic model are the RPA and GPA. RPA is based on 4 stratifying factors (Karnofsky Performance Status (KPS), age, extracranial distant metastases and controlled primary tumor), while GPA considers the amount of brain damage as an additional prognostic factor for BMS on the basis of RPA. RPA and GPA models are general prognostic models for BMs which are not directly concerned with lung cancer, unfortunately. In 2017, some researches [9] established a new prognostic model for NSCLC: Adjusted prognostic Analysis (APA) which includes new prognostic indicators smoking history and EGFR genotype. All previously published models have their respective advantages (easy to use and remember) and limitations (qualitative and subjective), and we hope to eliminate the components of other indicators that are difficult to quantify and/or subjective, such as the control of extracranial diseases. Therefore, it is necessary to focus on new markers and explore more reliable prognostic indicators to make up and update for the shortcomings of the existing prognostic models and improve the predictive value of clinical outcomes for NSCLC patients with BMs.

Blood biomarkers can be determined in a simple, quick, and stable manner, with negligible side effects to the patient, which commonly used to detect diagnostic, prognostic, and therapeutic decision-making markers of many cancers [10]. Inflammation is a tissue response to eliminate tissue damage. However, dysregulated inflammation is a recognized cause of cancer [11]. Numerous studies have shown that serum inflammatory indexes are closely related to the prognosis of lung cancer, such as alanine aminotransferase (ALT) [12, 13], Body Mass Index(BMI), serum albumin (ALB) [13, 14], prognostic nutritional index (PNI), systemic immune-inflammation index (SII) [15], lymphocyte count and Neutrophil to lymphocyte ratio(NLR) [16]. Nonetheless, few researchers have utilized a combination of clinical characteristics and serum inflammatory indexes in order to predict a prognosis of NSCLC patients with BMs.

Therefore, this study aims to establish a new prognostic model based on clinical characteristics and inflammation indicators by using LASSO-Cox regression analysis, so as to more accurately reflect the prognostic information of BMs in NSCLC patients comparing with APA, RPA and GPA. And to assess its incremental value in traditional prognostic models and provide a basis for clinicians to formulate reasonable treatment plans.

## Materials and methods

### Patient population and follow up

We retrospectively screened patients with BMs at the initial diagnosis of NSCLC who received treatment at Sun Yat-Sen University Cancer Center from January 2011 to December 2015. The eligibility criteria included: (1)pathologically confirmed and treatment naive NSCLC; (2) BMs confirmed by brain MRI; (3)available baseline clinical information, laboratory data, and all data collection prior to antitumor treatments; (4) patients did not suffer from any cancer disease prior to NSCLC diagnosis;(5) all deaths were cancer-related.

The survival data of each patient was obtained by reviewing medical records, emailing, and direct telecommunication. The last follow-up was conducted in April 2021. Overall survival (OS) was defined as the time interval from date of diagnosis to the date of patient’s death or last follow-up.

### Data collection

Baseline clinical characteristics were collected from the patients’ medical records patient characteristics (gender, age group, smoking history, KPS, BMI and family history), disease characteristics (tumor histology, location, extracranial distant metastases, number of brain metastatic lesions, EGFR mutation status), treatments (thoracic local treatment, EGFR-tyrosine kinase inhibitors (TKIs) treatment, Intracranial metastases local treatment, chemotherapy), previously published models information(RPA,GPA,APA), and the serum inflammatory indexes were collected included white blood cells (WBC), neutrophils (N), lymphocytes (L), platelets (PLT), NLR, platelet/lymphocyte ratio (PLR), derived neutrophil/lymphocyte ratio (dNLR), SII: SII was calculated by the formula: platelet(PLT) × neutrophil (N)/lymphocyte (L) [17], PNI: PNI was calculated by the formula ALB (g/L) + 5 × lymphocyte count × 10^9^/L [18], ALT, aspartate aminotransferase (AST), ALT/AST ratio (LSR), lactate dehydrogenase (LDH), ALB, C-reactive protein (CRP), ALB/CRP ratio (ACR). Advance lung cancer inflammation index(ALI): ALI was calculated by the formula: BMI × Alb/NLR [12]. Patients were randomized intodivided into Derivation cohort (70%) and Validation cohort (30%).

### Statistical analysis

Statistical analysis was conducted using R software (version 3.6.1). Continuous variables were represented as mean ± SD and analyzed using t-test or Wilcoxon test. Firstly, we utilized the LASSO-Cox regression algorithm, for which the λ value was determined by tenfold cross validation with the error of the minimum criteria to choose the most useful prognostic factors among all NSCLC-associated serum inflammatory indexes. Then a prognostic model was constructed to predict OS based on the coefficients of significant predictors that were derived from the LASSO-Cox regression. The following formula was used to calculate the risk score:

$${\text{Risk score}}\, = \sum_i^n\, Xi\, \times \,Yi$$

Subsequently, the predictive accuracy and discriminative ability of the novel prognostic model was compared with RPA,GPA, and APA using the Harrell concordance index (C-index), time-dependent ROC (td-ROC) curves [19], and Decision Curve Analysis(DCA) [20]. The larger C-index and area under the curve (AUC) of td-ROC curves, the better the model was for risk prediction. DCA make curves of different point cuts, pay attention to the relationship between benefits and risks brought by different point cuts in different models, and calculate the improvement after reclassification [21]. Pearson correlation coefficient was used to assess the correlation between the new prognostic model, RPA,GPA and APA. In addition, we developed a nomogram that integrates the prognostic model, RPA,GPA, and APA that may assist in individual survival prediction of NSCLC patients with BMs. Internal validation and calibration of the nomogram were performed using 1000-resample bootstrapping. Finally, we illustrated discrimination by dividing patients into low-risk groups and high-risk groups as per the novel predictive model scores. The Kaplan–Meier method was used to perform OS analysis. The log-rank test was utilized to compare significance of the differences of survival distribution between the groups. Generally, a p value ≤ 0.05 was considered statistically significant across all analyses.

## Results

### Patient characteristics

A total of 171 consecutive patients (121 in derivation cohort and 50 in validation cohort) with newly diagnosed BMs from NSCLC were enrolled. 94 (55.0%) of these patients were male, and 77 (45.0%) were female. The median age was 57 (inter quartile range [IQR], 51.0–62.0) years. The median follow-up for OS was 21 months. The 1-, 3-, and 5-year OS rates were 66.7%, 39.8%, and 30.4%, respectively. Only 81 patients (66.9%) in the derivation cohort had an identified EGFR genotype whereas more patients in the validation cohort had known EGFR (42, 84.0%) status. Most of other characteristics were similar between the two cohorts. The patients’ baseline characteristics are listed in supplement Table 1.

**Table 1:** The C-index of the our prognostic model, APA, RPA and GPA for prediction of OS in the derivation cohort and validation cohort

Factors	C-index (95% CI)	*P* value
For derivation cohort		
Our model	0.672 (0.609 ~ 0.736)	
APA model	0.597 (0.537 ~ 0.657)	
RPA model	0.517 (0.469 ~ 0.566)	
GPA model	0.514 (0.448 ~ 0.579)	
Our model vs APA model		0.049*
Our model vs RPA model		< 0.001*
Our model vs GPA model		< 0.001*
For validation cohort		
Our model	0.738 (0.657 ~ 0.819)	
APA model	0.637 (0.550 ~ 0.724)	
RPA model	0.520 (0.456 ~ 0.585)	
GPA model	0.634 (0.548 ~ 0.720)	
Our model vs APA model		0.024*
Our model vs RPA model		< 0.001*
Our model vs GPA model		0.052

*C-index* concordance index, *CI* confidence interval; P values are calculated based on normal approximation using function rcorrp.cens in Hmisc package. *P < *0.05*

### Construction of prognostic model for OS

**Fig. 1:**
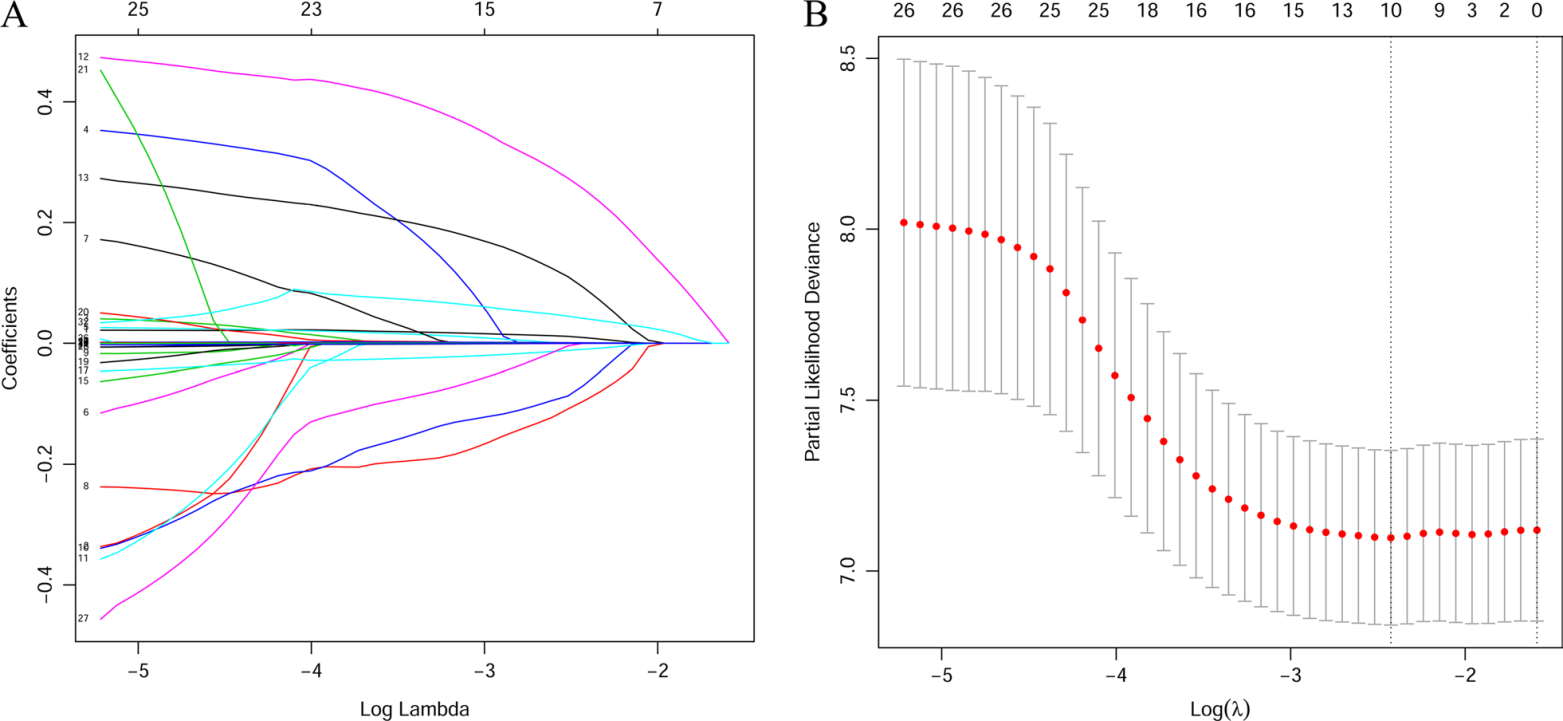
7value for λ was determined using tenfold cross-validation with the minimum criteria(Color should be used for any figures in print)

Firstly, LASSO-Cox regression analysis was carried out to extract significant predictors associated with OS in NSCLC patients with BMs. Figure 1A showed the analysis of the trajectory changes of each predictor variable. After, the optimal value for λ was determined through the use of tenfold cross-validation with minimum criteria (Fig. 1B). According to the criteria, the optimal value of λ in this study was 0.088. Its corresponding predictors were considered to be significant prognostic factors for OS, including age, Chemotherapy, TKIs, EGFR, Thoracic local treatment, ALB, ACR, LDH, ALI, and WBC. Finally, a prognostic model was constructed to predict OS based on the coefficients of the ten predictors that were derived from the LASSO-Cox regression. The following formula was used to calculate the risk score: the prognostic model risk score = "age"*0.0093—"Chemotherapy"*0.0950—"TKIs"*0.0690 + "EGFR"*0.2551 + "Thoracic local treatment"*0.0918 − "ALB"*0.0102 − "ACR"*0.0004 + "LDH"*0.0006 − "ALI"*0.0001 + "WBC" *0.0409. In the formula, the values of serum variables represent the respective serum original levels, and the code of clinical characteristic variables are listed in supplement Table 1.

### Assessing performance between the novel prognostic model, APA, RPA and GPA

**Fig. 2:**
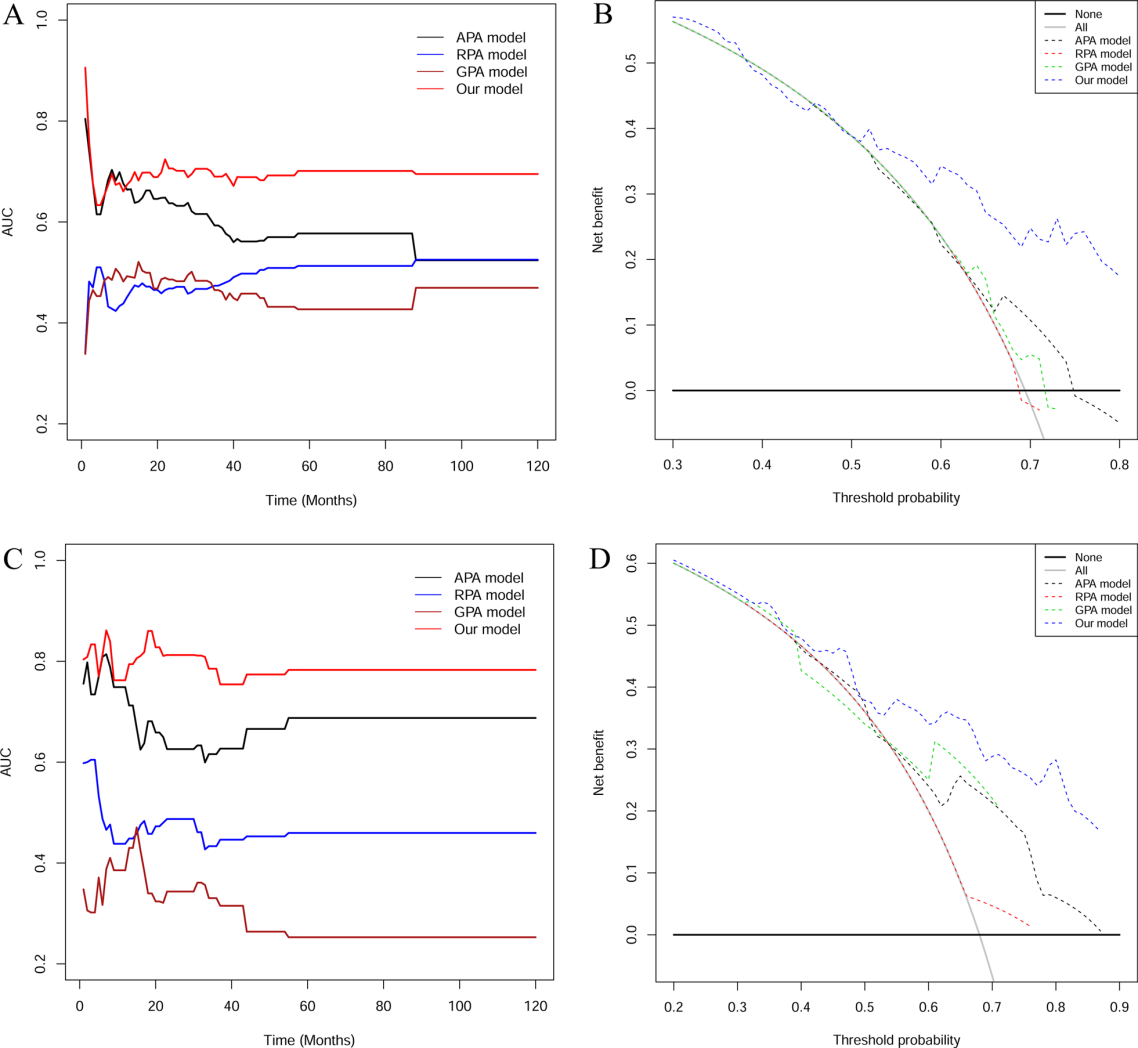
The results of AUCs and DCA in cohorts. The dynamic AUC levels of the four models in derivation cohort (**A**) and validation cohort (**C**). DCA for different prognostic models in derivation cohort (**B**) and validation cohort (**D**)

In order to evaluate the predictive values of the novel prognostic model for APA, RPA and GPA of OS, we introduced C-index, td-ROC curve, and DCA to evaluate the predictive accuracy of them. Firstly, we calculated the C-index of the four predictive model in the derivation cohort (Table 1). For OS, the C-index of our model was 0.672 (95% CI = 0.609 ~ 0.736), which was significantly higher than that of the APA [0.597 (95% CI = 0.537 ~ 0.657), p = 0.049], RPA [0.517(95% CI = 0.469 ~ 0.566), p < 0.001], and GPA [0.514 (95% CI = 0.448 ~ 0.579), p < 0.001]. Secondly, we plotted the td-ROC curves and calculated its corresponding AUCs. Results showed that AUCs of our model were higher compared to that of APA, RPA, and GPA with regards to OS at different time points (Fig. 2A). Thirdly, we drew graphs of threshold probabilities and net benefits for different prognostic models, and put them together to form a comparison of DCA for different prognostic models. Results demonstrated that the GPA and RPA curves are very close to the extremes and have little clinical value. APA's benefit is higher than the extreme curve, but it is still much lower than our model's which has a very high benefit over a wide threshold range. This means that compared with the old models, our model has the most practical implications for clinical practice (Fig. 2B). Similarly, we compared C-index (Table 1), AUCs (Fig. 2C) and DCA (Fig. 2D) in validation cohort and the results were consistent with the above.

Finally, both IDI and NRI calculations were utilized to compare alternative prognostic indices of our model with other models. Positive value represents better accuracy while negative value represents worse accuracy. The results were presented in Table 2. For derivation cohort, IDI analysis indicated that accuracy of our prognostic model was higher compared to that of the APA (0.138, p < 0.001), RPA (0.163, p < 0.001), and GPA (0.156, p < 0.001). NRI analysis indicated that accuracy of our prognostic model was higher compared to that of the APA (0.283, p < 0.001), RPA (0.283, p < 0.001), and GPA (0.388, p < 0.001). The results in validation cohort are similar to the derivation cohort (Table 2).

**Table 2:** The IDI and NRI were used to assess reclassification performance and improvement in discrimination of our novel prediction model

	IDI^a^	P Value	NRI^b^	*P* value
For derivation cohort				
Our model vs APA model	0.138	< 0.001*	0.283	< 0.001*
Our model vs RPA model	0.163	< 0.001*	0.283	< 0.001*
Our model vs GPA model	0.156	< 0.001*	0.388	< 0.001*
For validation cohort				
Our model vs APA model	0.139	0.158	0.272	0.178
Our model vs RPA mode	0.235	< 0.001*	0.460	0.020*
Our model vs GPA model	0.108	0.198	0.246	0.099

*IDI* integrated discrimination improvement index, *NRI* net reclassification improvement index. *P < 0.05

^a,b^Positive velue represents better accuracy, negative velue represents worse accuracy

### Construction of a predictive nomogram based on prognostic model, APA, RPA, and GPA

**Fig. 3:**
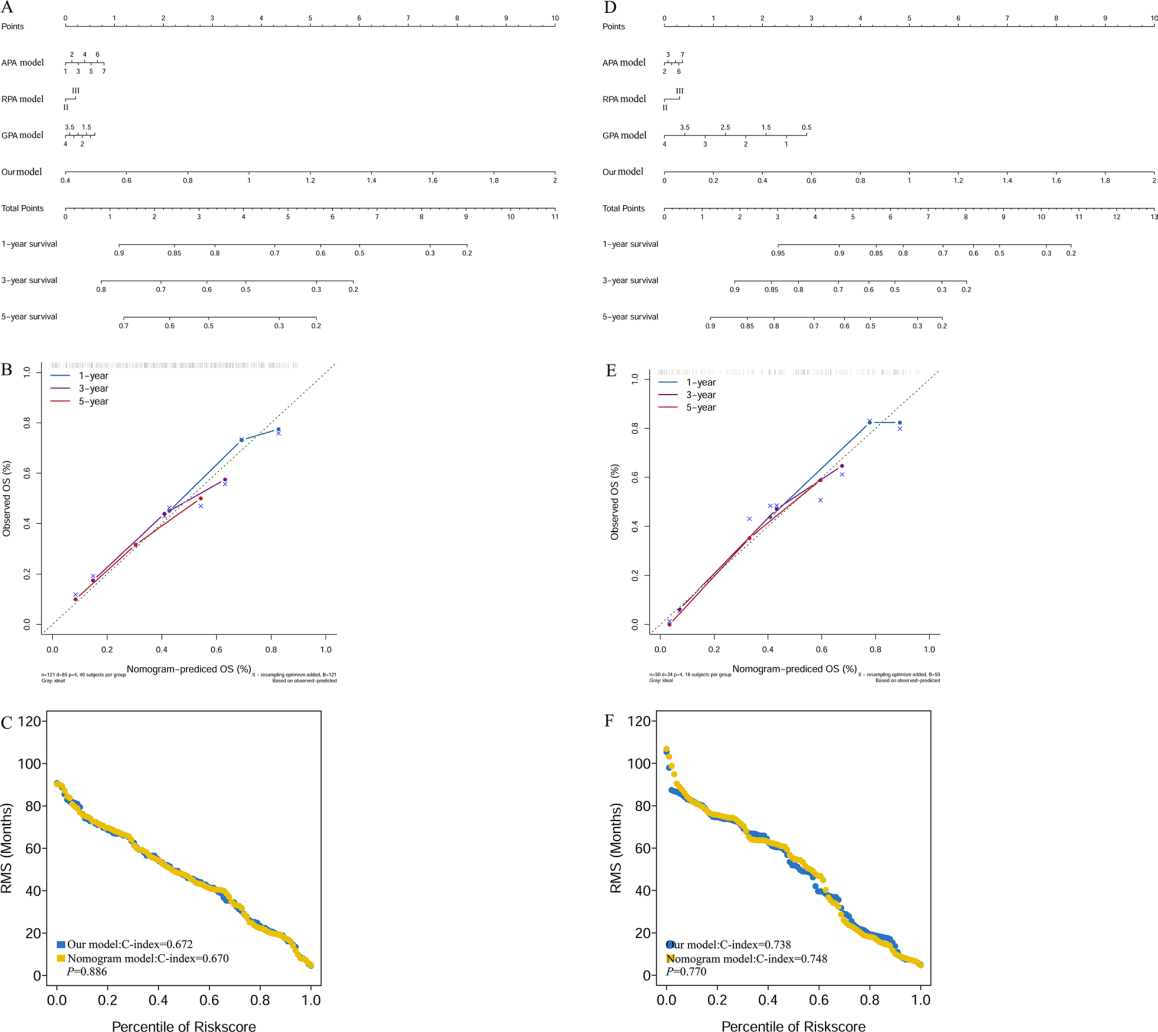
Construction of predictive nomogram and comparisons.The nomogram and calibration plots for estimating OS at 1, 3, and 5 years in derivation cohort (**A**, **B**), and validation cohort (**D**, **E**). The differences of C-index between nomogram model and our model in the derivation cohort (**C**) and validation cohort (**F**)

The nomogram incorporated the prognostic model, APA, RPA, and GPA to predict the probability of 1-, 3-, and 5-year OS (Fig. 3A) in the derivation cohort. Each patient was assigned a score for each prognostic variable. All scores were added together to estimate the probability of 1-, 3-, and 5- years OS. The higher the total score, the worse the patient’s prognosis. Besides, the calibration curve indicated good agreement between prediction and observation in 1-, 3-, and 5-year OS (Fig. 3B). Furthermore, we calculated the C-index of all predictive models in the derivation cohort. The results showed that the prognostic model has the same predictive value as nomogram models (p = 0.886, Fig. 3C). The differences of C-index between other models in derivation cohort are detailed in subTable 1. Similarly, we construct nomogram model of the validation cohort (Fig. 3D) and analyzed the calibration curves of nomogram model (Fig. 3E) and compared C-index between nomogram model and our model (Fig. 3F). The results are similar to the derivation cohort. The comparison of C-index among all the five models in validation cohort is shown in supplement Table2.

### The correlation between the novel prognostic models and other models

**Fig. 4:**
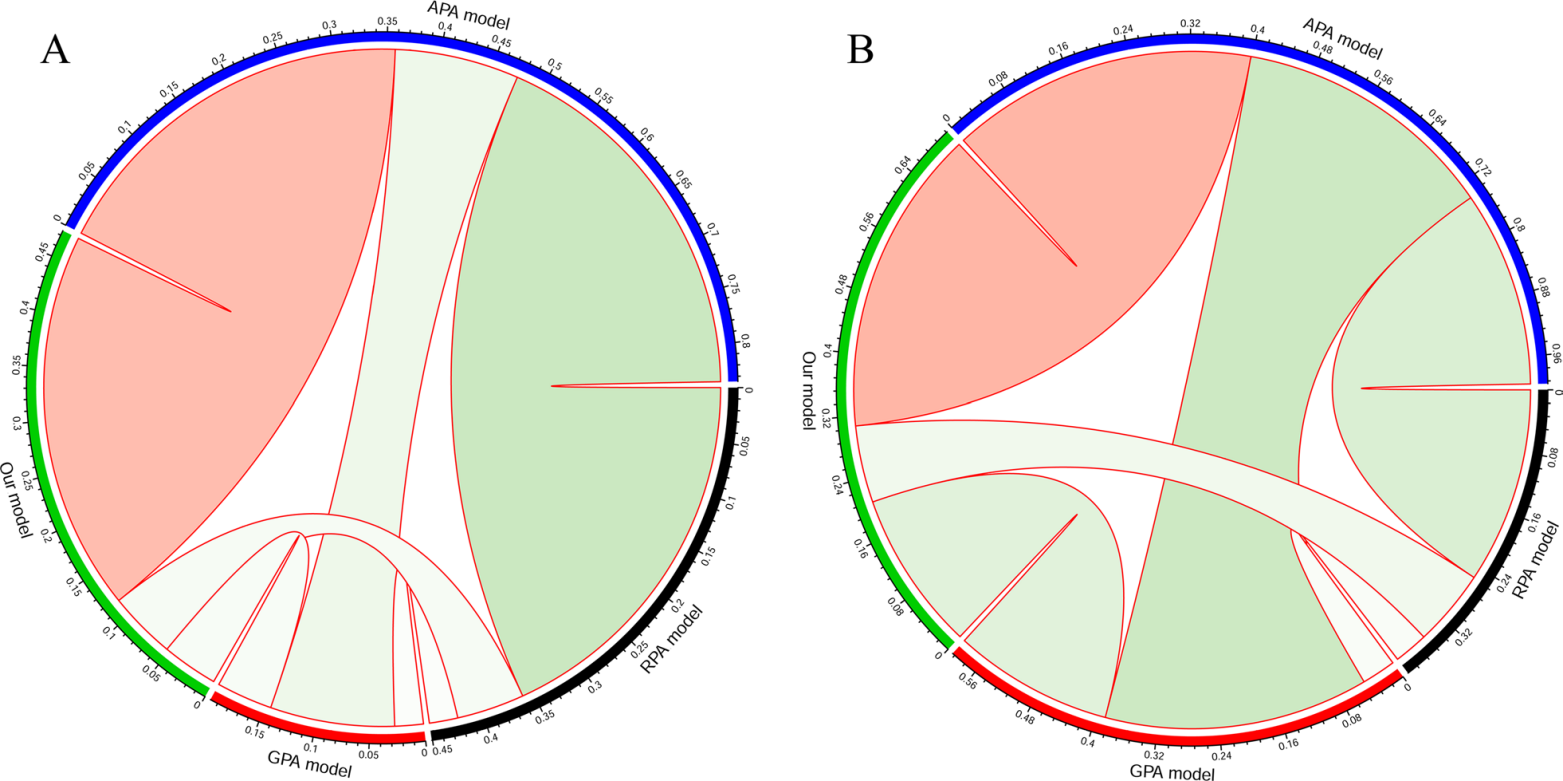
The correlations between the prognostic model, APA, RPA, and GPA. The red represented positive correlation and the green represented negative correlation. Significant linear dependence between variables was identified using Pearson's correlation coefficient (PCC)

We evaluated the correlation between the prognostic model and other models (Fig. 4). In this plot, the green represented negative correlation and red represented positive correlation. The size of a circle and color intensity were directly proportional to the correlation coefficients. We utilized Pearson's correlation coefficient to determine a significant linear correlation between the variables. The results demonstrated that our prognostic model was positively correlated with APA (correlation coefficient = 0.357, p < 0.001) while with other models were negatively correlated in derivation cohort. There were same correlation trends and differences in validation cohort showed in Table 3.

**Table 3:** The correlation between our model and other models

Models	Correlation coefficients ^a^	*P* value
For training cohort		
Our model vs APA model	0.357	< 0.001*
Our model vs RPA model	− 0.064	0.486
Our model vs GPA model	− 0.053	0.564
For external validation cohort		
Our model vs APA model	0.398	0.004*
Our model vs RPA model	− 0.100	0.490
Our model vs GPA model	− 0.208	0.148

^a^Pearson's correlation coefficient. *P < 0.05

### Survival analyses according to the prognostic model, APA, RPA, and GPA

**Fig. 5:**
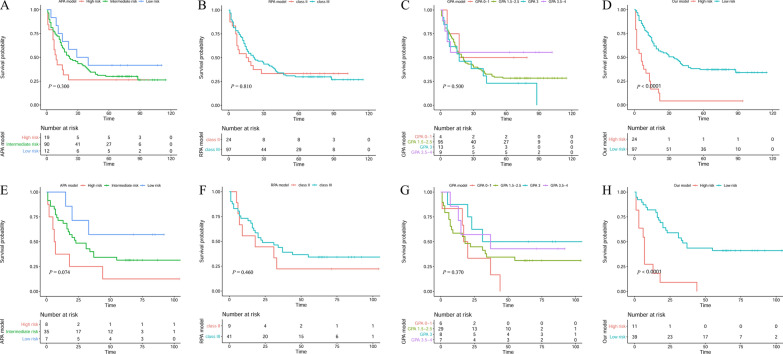
Kaplan–Meier analysis in different models. APA, RPA, GPA, and our prognostic model in derivation cohort (**A**–**D**) and in validation cohort (**E**–**H**)

We classified patients into low-risk patients and high-risk patients based on the prognostic model risk score and made the Kaplan–Meier curve for our prognostic model. The APA, RPA, and GPA were grouped according to their respective classification methods. In the derivation cohort, the results indicated that patients with higher risk scores (risk score > 1.33) had a significantly lower OS (Fig. 5D; p < 0.001) rate compared to the low-risk (risk score ≤ 1.33) counterparts. So did the validation cohort (Fig. 5H, p < 0.001). However, patients could not be effectively distinguished between different risk groups in derivation cohort (Fig. 5A–C) and in validation cohort (Fig. 5E–G) based on APA, RPA, and GPA. The results indicated that our prognostic model had improved performance in distinguishing the prognosis of patients in NSCLC with BMs than others.

## Discussion

In this study, we analyzed the clinical characteristics and serum inflammatory indexes of individuals based on survival analysis to establish a novel prognostic model to predict OS in NSCLC patients with BMs by using LASSO-Cox regression algorithm. Compared to traditional prognostic model APA, RPA, and GPA, our prognostic model had improved prediction accuracy and ability to distinguish between the groups. Our prognostic model successfully classified those patients into high-risk and low-risk subgroups, which were significantly different with regards to OS.

Algorithm LASSO-Cox regression analysis has been applied across many studies as a statistical method because of adjusting the model’s over fitting to avoid extreme predictions and significantly improving the prediction accuracy [22–24]. In this research, we utilized the new algorithm LASSO-Cox regression analysis, we identified a ten individual prognostic factors (some were reported and others were new) and incorporated them into a novel predictive model to predict OS in NSCLC patients with BMs. Age is a factor common to all four models, while chemotherapy, TKIs, and EGFR mutation status are specific to APA. This may be explained by the specificity of APA focusing on predicting patients in NSCLC with BMs, while other models are common to all kinds of tumors with BMs. In our model, we identified six new individual prognostic factors (thoracic local treatment, ALB, ACR, LDH, ALI, and WBC) which had not been considered in all those previously published prognosis models. Although surgery is the main treatment for early lung cancer, it had been reported that surgery for the primary tumor was associated with a superior patient outcome in NSCLC with BMs [25].

In 2013, Jafri SH et al. proposed a new prognostic index ALI for patients with advanced lung cancer [12]. ALI is combined with BMI, plasma ALB, and NLR [16, 26, 27]. Previous studies have shown that ALI has certain prognostic value for patients with esophageal cancer, lung cancer and malignant lymphoma [28]. BMI and serum ALB are not only represent the nutritional status of patients to some extent, but also closely related to tumor progression [13]. Besides, malnutrition is positively correlated with the decrease of quality of life score and the severity of patients' symptoms [14]. Carcinogenesis is a complex, stepwise process that involves the acquisition of genetic mutations and epigenetic changes [29], the environmental and hereditary factors, stochastic effects [30] and inflammation [31, 32]. Rodents animal studies have revealed that chronic inflammation significantly enhances lung carcinogenesis, and inhibition of inflammation suppresses cancer progression and reduces the tumor volume [33]. Besides, the role of inflammation in increasing the risk of lung tumorigenesis driven primarily by oncogenic KRAS has been researched and the results showed that inflammatory responses may increase KRAS mutation rate and create a vicious cycle of chronic inflammation and KRAS mutation [34]. Fortunately, many studies have clarified the molecular mechanism and roles of chronic inflammation in lung cancer [35] and various immune cells, cytokines and signaling pathways participate in inflammation mediated lung carcinogenesis [33]. Acute lung inflammation is dominated by neutrophils, whereas chronic reactions mainly involve macrophages and lymphocytes [35], so it is easy to understand the predictive value of WBC in cancers. In 2021, Berghoff [36] et al. also reported inflammatory markers were associated with OS in patients with newly diagnosed brain metastases. Compare to that study, we focused on the prognosis of patients with BMS in NSCLC only, making the study more targeted for clinical application. More importantly, our study is a quantitative prognostic risk model jointly constructed by multiple indicators, which is convenient for doctors to conduct personalized assessment and treatment of patients.

Increasing uptake of glucose and preferential conversing of glucose to lactate are a generic feature in types of cancer although the precise role of the Warburg effect [37] is not fully understood. LDH is the enzyme responsible for conversion of pyruvate to lactate at the endpoint of glycolysis. LDH regulates the rapid growth of tumor cells and makes the disease progress [38–40]. Studies have shown that elevated serum LDH concentration can lead to poor prognosis of lung cancer patients with different pathological types [41–46] and be useful in monitoring of treatment in advanced NSCLC [47]. The preoperative LDH concentration and postoperative LDH concentration change trend were independent prognostic factors for patients in lung large-cell neuroendocrine carcinoma (L-LCNEC) [48]. Based on the evidence above, LDH as a predictor in our model is valid and credible.

Compared to previous models, the novel model had several advantages. Including more potential prognostic factors in which serum inflammatory factors are mentioned for the first time is the most striking feature in our study. This method significantly improves the accuracy compared with traditional COX regression analysis. However, there were still some limitations in this study. Selection bias may be unavoidable in all retrospective analysis, and especially in this single cancer center with a small sample size relatively. So it is necessary to carry out multi-center and large-scale studies in the future to further verify the generalizability of our prognostic model established in this study. Although these predictors in our model were easy to obtain, it was undeniable that they were all non-specific predictors for NSCLC with BMs. NSCLC-related immunohistochemical markers [49] may be incorporated into prognostic models to improve specificity, such as PD-1 [50–53], EML4-ALK [54–58], and VEGF [59–61]. In addition, we established the model with initial diagnosis data, so we could not know the prognosis of the patient after each treatment. We can also collaborate clinically in the future, focusing on the establishment of prognostic models related to treatment duration.

## Conclusions

In summary, we established a novel prognostic model successfully based on clinical characteristics and serum inflammatory factors which outperformed APA, RPA, and GPA in predicting OS in NSCLC patients with BMs. This prognostic model may act as a potential tool for clinicians to provide consultation, personalized treatment and follow-up for NSCLC patients with BMs due to the low cost, easy operation, precision, and stability. However, the wide practical application of this model required more clinical data and multi-center verification to verify the accuracy of our model in predicting prognosis of NSCLC patients with BMs.

## Supplementary Information

**Additional file 1: Table S1**. Clinical characteristics of derivation and validation cohorts.

## Data Availability

The datasets analysed during the current study are available in the Research Data Deposit public platform (www.researchdata.org.cn) provided by RDD Management Committee in the Sun Yat-sen University Cancer Center.

## Abbreviations

NSCLC: Non-small cell lung cancer;BMs: brain metastases;
C-index: Concordance index
td-ROC: Time-dependent receiver operating characteristic curve
KPS: Karnofsky Performance Status
DCA: Decision Curve Analysis
NRI: Net reclassification improvement index
IDI: Integrated discrimination improvement index
BMI: Body Mass Index
EGFR: Epidermal Growth Factor Receptor
TKIs: EGFR-tyrosine kinase inhibitors
APA: Adjusted prognostic Analysis
RPA: Recursive Partition Analysis
GPA: Graded Prognostic Assessment
WBC: White blood cells
NLR: Neutrophil/lymphocyte ratio
PLR: Platelet/lymphocyte ratio
dNRI: Derived neutrophil/lymphocyte ratio
SII: Systemic immune-inflammation index
PNI: Prognostic nutritional index
ALB: Albumin
ALT: Alanine aminotransferase
AST: Aspartate aminotransferase
LSR: ALT/AST ratio
LDH: Lactate dehydrogenase
CRP: C-reactive protein
ACR: ALB/CRP ratio
ALI: Advance lung cancer inflammation index
