# Supplementary material for: A novel quantitative prognostic model for initially diagnosed non-small cell lung cancer with brain metastases
Source: Cancer Cell Int. 2022 Aug 11;22:251. doi: 10.1186/s12935-022-02671-2 (PMC9367158; doi:10.1186/s12935-022-02671-2)
Supplement: Supplementary file 1 — Additional file 1: Table S1. Clinical characteristics of derivation and validation cohorts. [file 12935_2022_2671_MOESM1_ESM.doc]

supplementTable 1. Clinical characteristics of derivation and validation cohorts

| Characteristics |  | Code^#^ | Derivation cohort(n=121) | Validation cohort(n=50) | *P* value |
| --- | --- | --- | --- | --- | --- |
| EGFR mutation status | Wild type | 0 | 44(36.4%) | 15(30.0%) | 0.010* |
|  | Mutation type | 1 | 37(30.6%) | 27(54.0%) |  |
|  | unknown | 2 | 40(33.1%) | 8(16.0%) |  |
| Thoracic local treatment | Surgery | 1 | 10(8.3%) | 2(4.0%) | 0.391 |
|  | Radiotherapy | 2 | 2(1.7%) | 0(0.0%) |  |
|  | Not received | 3 | 109(90.1%) | 48(96.0%) |  |
| TKIs | Not received | 0 | 97(80.2%) | 38(76.0%) | 0.543 |
|  | Received | 1 | 24(19.8%) | 12(24.0%) |  |
| Chemotherapy | Not received | 0 | 37(31.6%) | 17(34.0%) | 0.662 |
|  | Received | 1 | 84(69.4%) | 33(66.0%) |  |
| Age (years) | x±S | original | 55.8±9.7 | 57.7±8.8 | 0.244 |
| ALB | x±S | original | 41.2±4.1 | 40.6±3.9 | 0.223 |
| ACR | x±S | original | 22.0±33.3 | 12.6±17.1 | 0.203 |
| LDH | x±S | original | 239.2±120.6 | 266.3±165.2 | 0.734 |
| ALI | x±S | original | 308.0±165.9 | 317.7±177.8 | 0.857 |
| WBC | x±S | original | 8.8±3.0 | 8.9±3.8 | 0.753 |
| Gender | Male | / | 66(54.5%) | 28(56.0%) | 0.862 |
|  | Female | / | 55(45.5%) | 22(44.0%) |  |
| KPS | >70 | / | 115(95.0%) | 46(92.0) | 0.680 |
|  | ≤70 | / | 6(5.0%) | 4(8.0%) |  |
| Smoking history | Never-smoker | / | 73(60.3%) | 24(48.0%) | 0.109 |
|  | ever-smoker | / | 46(39.7%) | 26(52.0%) |  |
| [Family](javascript:;) [history](javascript:;) | No | / | 96(79.3%) | 39(78.0%) | 0.845 |
|  | Yes | / | 25(20.7%) | 11(22.0%) |  |
| Location | Upper | / | 59(48.8%) | 18(45.0%) | 0.312 |
|  | Lower | / | 60(49.6%) | 31(53.2%) |  |
|  | Other |  | 2(1.7%) | 1(1.8%) |  |
| Tumor histology | Adenocarcinoma | / | 96(81.0%) | 37(74.0%) | 0.685 |
|  | Squamous | / | 8(5.0%) | 5(10.0%) |  |
|  | Other | / | 17(14.0%) | 8(16.0%) |  |
| Intracranial metastases local treatment | WBRT | / | 5(4.1%) | 11(22.0%) | 0.000 |
|  | surgery | / | 0(0.0%) | 0(0.0%) |  |
|  | SRT | / | 9(7.4%) | 0(0.0%) |  |
|  | Not received | / | 107(88.5%) | 39(78.0%) |  |
| Number of brain metastatic lesions | single | / | 45(37.2%) | 18(36.0%) | 0.883 |
|  | Multiple | / | 76(62.8%) | 32(64.0%) |  |
| Extracranial distant metastases | No | / | 32(26.4%) | 17(34.0%) | 0.320 |
|  | Yes | / | 89(73.6%) | 33(66.0%) |  |
| APA | High risk | / | 19(15.7%) | 8(16.0%) | 0.731 |
|  | Intermediate risk | / | 90(74.4%) | 35(70.0%) |  |
|  | Low risk | / | 12(9.9%) | 7(14.0%) |  |
| GPA | 0-1 | / | 4(3.3%) | 6(12.0%) | 0.029* |
|  | 1.5-2.5 | / | 95(78.5%) | 29(58.0%) |  |
|  | 3 | / | 13(10.7%) | 8(16.0%) |  |
|  | 3.5-4 | / | 9(7.4%) | 7(14.0%) |  |
| RPA | Class II | / | 24(19.8%) | 9(18.0%) | 0.782 |
|  | Class III | / | 97(80.2%) | 41(82.0%) |  |
| BMI | <18.5 kg/m^2^ | / | 15(12.4%) | 9(18.0%) | 0.107 |
|  | 18.5–22.9 kg/m^2^ | / | 65(53.7%) | 18(36.0%) |  |
|  | ≥23.0 kg/m^2^ | / | 41(33.9%) | 23(46.0%) |  |
| CRP | x±S | / | 16.2±24.1 | 21.1±31.3 | 0.219 |
| ALT | x±S | / | 24.1±16.5 | 22.9±14.6 | 0.769 |
| AST | x±S | / | 21.8±8.6 | 23.0±16.4 | 0.991 |
| LSR | x±S | / | 1.1±0.4 | 1.7±0.7 | 0.252 |
| [Lymphocyte](javascript:;) | x±S | / | 1.8±0.7 | 1.8±0.6 | 0.924 |
| N[eutrophil](javascript:;) | x±S | / | 6.2±2.6 | 6.4±3.7 | 0.539 |
| PLT | x±S | / | 275.1±98.4 | 268.7±91.8 | 0.491 |
| NLR | x±S | / | 3.9±2.5 | 4.1±3.0 | 0.687 |
| dNLR | x±S | / | 2.6±1.6 | 2.9±2.5 | 0.777 |
| PLR | x±S | / | 172.6±93.9 | 167.5±85.8 | 0.547 |
| SII | x±S | / | 1114.3±949.7 | 1112.2±931.5 | 0.727 |
| PNI | x±S | / | 50.1±5.8 | 49.4±5.1 | 0.495 |

TKIs :EGFR-tyrosine kinase inhibitors (TKI) treatment; ALB: albumin; ACR: ALB / CRP ratio; LDH: lactate dehydrogenase; ALI :Advance lung cancer inflammation index;WBC: white blood cells; KPS: Karnofsky Performance Status; APA: Adjusted prognostic Analysis; GPA : Graded Prognostic Assessment ; RPA: Recursive Partition Analysis ;BMI: Body Mass Index; CRP: C-reactive protein ;ALT: alanine aminotransferase; AST: aspartate aminotransferase ;LSR: ALT / AST ratio; PLT: platelet; NLR: neutrophil / lymphocyte ratio ;dNLR: derived neutrophil / lymphocyte ratio; PLR: platelet/lymphocyte ratio; SII: systemic immune-inflammation index ;PNI: prognostic nutritional index.

^#^ Codes are only used in formula which contains 10 predictors on the top of list.

**P*<0.05.
